# A Community Orthopaedic Residency Approach to Education and Training During the COVID-19 Pandemic

**DOI:** 10.5435/JAAOSGlobal-D-20-00107

**Published:** 2021-01-12

**Authors:** Chris C. Oguayo, Jennifer Chu, Alan L. Jones

**Affiliations:** From the Baylor Scott & White, University Medical Center, Dallas, TX (Dr. Oguayo, Dr. Chu, and Dr. Jones); and Orthopedic Associates of Dallas, Dallas, TX (Dr. Oguayo, Dr. Chu, and Dr. Jones).

## Abstract

The COVID-19 pandemic has rapidly affected all facets of everyday life including the practice of medicine. Hospital systems and medical practices have evolved to protect patients, physicians, and staff and conserve personal protective equipment and resources. Orthopaedic practices have been specifically affected by social distancing and stay at home guidelines, limiting in-office practice and elective surgery restrictions. This, in turn, has had an effect on resident education. Previous literature has been published regarding how academic programs have adjusted to these changes. However, the effects on smaller orthopaedic residencies with nonacademic faculty has not been discussed. The orthopaedic residency at Baylor University Medical Center of Dallas is a fifteen-resident program with a combination of hospital employed and private practice faculty. We adjusted our resident education in mid-March 2020 to keep residents safe while trying to maximize surgical and clinical education and outside research.

**Goals:**

Our goals were to come up with a plan allowed for continuing high-level patient care and resident education while protecting residents and limiting burnout.

**Model:**

We devised a four-team system with five-day call periods. Interactions between teams were strictly minimized. We also moved to a web-based academic curriculum and devised a system for safe resident participation in surgical cases. The model has been adjusted based on attending and resident feedback.

**Conclusion:**

Until we develop effective treatments or vaccination for COVID-19, there is a possibility that it will be an ongoing threat. Resident education must also adapt to the changing environment while continuing to provide residents safe opportunities for patient care, didactic education, and research. We believe we have come up with a sustainable, adaptable model for resident education during this challenging time.

COVID-19, named for a new coronavirus strain discovered in 2019,^1^ has rapidly changed all facets of our lives. During this pandemic, healthcare systems initially limited outpatient clinic interactions and deferred nonurgent or emergent surgeries indefinitely.^2^ Limiting interactions and adhering to social distancing guidelines seems to have a positive role in limiting the spread of COVID-19.^3^ However, these changes required immediate adjustments to resident education. Previous literature has been published regarding how traditional academic programs have adjusted to these changes.^4^ The orthopaedic residency program at Baylor University Medical Center Dallas is a community program that consists of three residents per year. Residents rotate through multiple facilities in the Dallas-Fort Worth area and spend a large portion of their nontrauma rotation in outpatient clinics. The faculty for the residency consists of a combination of hospital-employed physicians and private practice physicians. Because of the heterogeneous nature of the faculty, different adjustments were made by different practices in relation to the pandemic. In addition, because the residents rotate in different counties, local restrictions and guidelines also affected orthopaedic practice in distinct ways. As practice changes began in mid-March 2020, our program needed to make adjustments to safely provide ongoing care at a Level 1 trauma care and limit resident exposure while also offering residents continued opportunities to participate in surgery with nontrauma faculty. We will describe our plan along with the adjustments that have been made as restrictions have changed.

## Aims

Our goals when making a plan included the following:

1. Providing safe patient care with contingency plans in place in case of resident exposure
2. Maximizing safe opportunities for participation in surgery
3. Maintaining resident education through interactive didactics
4. Limiting resident burnout by giving residents regular updates and allowing them to participate in decisions regarding model changes

## Dividing the Residency

On March 23, 2020, we divided the residents into four separate care teams. We chose four teams because we have four full-time traumatologists on staff. This was done after discussion with core faculty members on the elective rotations including hand and upper extremity, foot and ankle, sports, and adult reconstruction. All residents from these rotations were pulled off their elective rotations to participate in the care teams. Interns who were on elective rotations were also pulled. The only residents who remained on their assigned rotations included a PGY3 on a pediatric rotation and an intern on general surgery. The rest of the residents were divided into groups of 2 to 3 along with the four orthopaedic trauma faculty on staff. Allocation was done through a random number generator with the senior residents and junior residents assigned separately. No specific reason was available for the randomization.

We decided on five-day rotations for each team because this gave approximately a two-week period between call cycles. The on-call team also covered hand surgery and spine surgery when a faculty member affiliated with the residency was on call. Each resident took one 24-hour shift of call with the ability to go home if no pending consults were available. A separate resident was available for back up in case of multiple surgeries (ie, trauma and hand) running simultaneously. Each resident was given pagers for each covered service. Multiple pagers were used when available to limit potential risk during hand-offs. When this was not possible, instructions were given for disinfecting pagers and leaving them at a designated location known to all residents. Sign-outs were done via either telephone or video chat applications. The members on a specific team also continued to round on the group of patients they cared for after their five call days ended.

**Figure 1:**
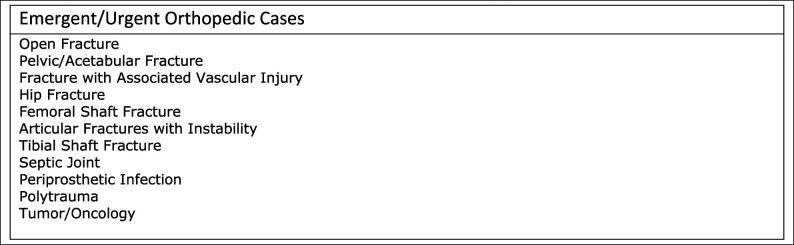


For surgical cases, consents were done via two separate copies of consent forms because COVID-19 may live on surfaces for hours or even days,^5^ and usually multiple people handle these forms. One is given for the patient to sign and is kept by the patient, whereas the other is signed by the physician and kept in patient records. The consent of record was maintained in the EMR electronically. For the month of April, all surgical cases were approved by the department chair. Figure 1 details a list of common procedures deemed emergent/urgent. No delays secondary to operating room or staff availability were noted because elective surgeries were not permitted at the hospital from March 16 to April 27. Each resident was given an N95 mask that is to be worn during the entirety of the case with a protective regular surgical mask over it. N95 masks and protective equipment were conserved and reused after decontamination, which was recommended to be done daily, is done via multiple methods, i.e., UV light and heat disinfection.^6-8^ Residents and attending physicians were not present in the operating rooms during the intubation or extubation process. Eye protection in the form of visors, eye and face shields, were available for resident use as well. The resident who was a part of the surgical case usually followed the patient until discharge or no longer needed orthopaedic care in the hospital.

After the resident's 5-day rotation, they were off service with the exception of rounding on patients for the next 15 days. Rounding times when not on call were at the discretion of the resident and attending physician. Efforts were made not to use the residents who had been in the hospital for outside cases for 7 days from their last on service day.

If a resident was exposed or experienced fever or respiratory symptoms potentially suggestive of COVID-19,^9-11^ the plan was for removal and replacement by a team member from a different rotation. All residents were notified if this occurred because it could affect their schedule. In addition, although we did not have to use this plan, a plan in place for removal of a whole team in case of exposure and replacing them with another team was available. Because the average incubation time is 5.1 days with most patients showing symptoms by day 12, a 15-day off period seemed reasonable for quarantine.^12^

To give residents the opportunity to participate in urgent cases being performed at outside hospitals by nonorthopaedic trauma faculty, a forum for regular communication, via electronic group chats and group e-mail, from core faculty regarding these cases was set up. Cases were mostly covered by the team who was next for call. Throughout the month of April, only one resident participated in each case.

**Figure 2:**
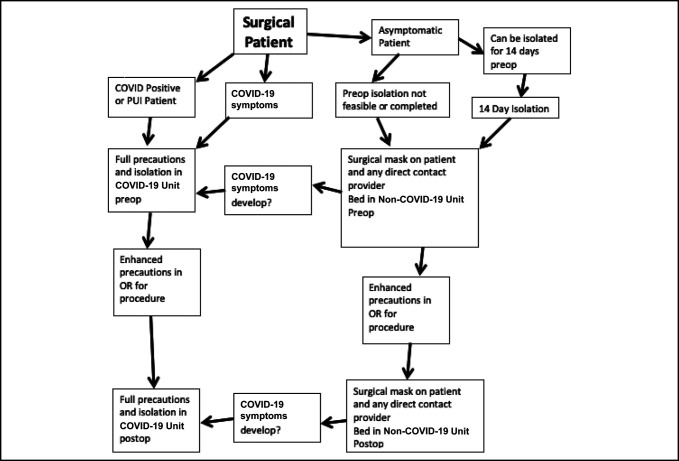


Our department also made efforts to limit exposure by coming up with an algorithm and protocol regarding patients who required surgery and were COVID-19-positive or had a high risk without time for testing (Figure 2). This is encompassed in a general algorithm of how we will evaluate surgical patients until the current threat decreases. Patients who can have definitive treatment delayed without having severe adverse effects and who are not disabled by their injury are deferred to outpatient orthopaedic surgeons. They are requested to self-quarantine for a 14-day period before definitive surgery. If they have already been in quarantine for 2 weeks, then they can proceed with surgery at the discretion of the treating orthopaedic surgeon. Patients who were either COVID-19-positive or with symptoms suggestive of COVID-19 are placed on COVID-19 units in the hospital, whereas those who do not meet those criteria are placed on regular ward floors. There are also COVID-19-specific operating rooms and postanesthesia care unit. This is not a perfect system and has inherent flaws which include

1. False positive COVID-19 tests
2. False negative COVID-19 tests
3. Exposing someone with COVID-19 symptoms not due to COVID-19 to a COVID-19 floor
4. Exposing a non-COVID-19 floor to an asymptomatic patient with COVID-19

Despite these flaws, the goal with this protocol is to deliver appropriate patient care while mitigating risk to staff. At this time, no residents are to be a part of surgical cases with known or suspected patients with COVID-19, and telemedicine evaluations are to be done unless there is a specific task that must be done in person by an orthopaedic surgeon, in which case the attending is notified.

## Resident Education

On March 16, 2020, our education moved to an entirely video-based curriculum. All in-person conferences remain suspended. However, most lectures and curriculum have continued. There are daily readings and questions as well as weekly peer-reviewed articles that every resident has access to. On Mondays, the 3 hours of weekly curriculum including attending lectures have continued on a video-based forum. All residents with the exception of the two who were not in the team-based rotations have participated. Journal club has also been held on a video-based forum. This time was also used for more casual discussion of how we were coping with the pandemic, changes in curriculum, and any new hobbies or goals we were currently working on. Our program chair and associate program director maintain an open-door policy if any issue or concern should arise.

## Future Changes

Because elective surgery restrictions in the state of Texas have changed, we are making plans to transition out of the team-based rotation. We are also tracking which residents had specific rotations affected by the pandemic and making efforts to find them opportunities to get exposure to these subspecialties in future years. As we look to the future, although, we continue to keep the four-team model available as a back-up, should the pandemic conditions worsen.

## Conclusion

The COVID-19 pandemic quickly changed the Baylor University Medical Center Dallas residency in the month of March and will likely continue to change the practice of orthopaedic. Using the Spanish Flu of 1918 as a model of how COVID-19 might behave, we have to anticipate multiple waves.^13^ We have described our experience in the months of March and April to show how our program tried to continue offering high-quality patient care while maintaining educational activities.
